# T3 Endoscopic Thoracic Ganglionectomy Using Cutting Mode Cautery for Palmar Hyperhidrosis

**DOI:** 10.1016/j.atssr.2024.12.004

**Published:** 2024-12-13

**Authors:** Joel Talsma, Crystal C. Peirce, Ivo Tarfusser, Subin Kim, Olajire Idowu, Sunghoon Kim

**Affiliations:** 1 Department of Biomedical Sciences, University of New England College of Osteopathic Medicine, Biddeford, Maine; 2 Department of Surgery, Villa Sant’Anna Clinic, Merano, Italy; 3 Department of Surgery, University of Kentucky Markey Cancer Center, Lexington, Kentucky; 4 Division of Pediatric Surgery, University of California San Francisco Benioff Children’s Hospitals, Oakland and San Francisco, CA

## Abstract

**Background:**

Understanding the precise anatomy of the upper thoracic sympathetic trunk is crucial for effectively treating palmar hyperhidrosis using the surgical technique of endoscopic thoracic sympathectomy (ETS). The variability in the location of T2 and T3 ganglia in relation to the ribs may contribute to inconsistent clinical outcomes after ETS.

**Methods:**

For the cadaver study, adult human cadaveric thoraces were dissected to map the locations of T2 and T3 ganglia by identifying their white rami communicantes. For the ETS group, T3 ganglionectomy ETS was performed. Surgical outcome and postoperative incidence of compensatory sweating were assessed.

**Results:**

A total of 74 pediatric patients underwent thoracoscopic T3 ganglionectomy. All patients were satisfied with the surgical outcomes. No patients complained of compensatory sweating. Forty cadavers were dissected: 25 adult female and 15 adult male. The findings showed that a high percentage of T2 ganglia are near the superior border of the third rib, while the T3 ganglion is near the fourth rib.

**Conclusions:**

Because of the proximity of the T2 ganglion to the third rib, accidental thermal injury to the T2 ganglion may occur when third rib-oriented ETS is performed. A ganglion-oriented T3 ETS can be accomplished by using the white ramus communicans as a guide to accurately locate the T3 ganglion.

**In Short**

▪ T2 ganglion lies near or on the third rib.
▪ Sympathectomy at rib 3 may cause thermal injury to the T2 ganglion.
▪ T3 ganglionectomy using cutting mode cautery can be performed by using the white ramus communicans as a guide to locate the T3 ganglion.

An accurate understanding of the anatomy of the upper thoracic sympathetic trunk is paramount to successfully treating palmar hyperhidrosis using endoscopic thoracic sympathectomy (ETS). The variable clinical outcomes after ETS for palmar hyperhidrosis may be due to not considering the exact location of the T2 and T3 ganglia in relation to their corresponding ribs.

As viewed with the videoscope, the T2 and T3 ganglia locations are not easily discernible; ganglia do not appear as plump round structures. For this reason, a rib-oriented endoscopic sympathectomy approach has been recommended.^1^ In other words, the second, third, and fourth ribs are used as guideposts rather than the actual positions of ganglia to determine the limits of sympathectomy. This approach may interrupt the sympathetic chain in random locations irrespective of the actual ganglia positions. Furthermore, it may lead to ganglia that are incompletely destroyed or sustain thermal injury.

For treating palmar hyperhidrosis, the level of thoracic ganglionectomy that will result in optimal clinical outcome is the T3 ganglion.^2^ Sympathetic innervations to the palm pass through and arise from T4, T3, T2, and T1 ganglia within the sympathetic trunk. To achieve a normal level of palm perspiration and to preserve sympathetic innervation to the heart, any thermal injury to the T2 ganglion should be avoided. By interrupting the sympathetic signals from T3 and T4 but preserving the sympathetic supply to the face from T1 and T2, the risk of compensatory sweating can be lowered while still achieving the desired clinical outcome.

The position of the T3 ganglion along the thoracic sympathetic trunk can be located by finding the white ramus communicans (RC) that comes off the third intercostal nerve (ICN) and following the ramus toward the sympathetic trunk. It is known that the junction where the white ramus joins the sympathetic trunk is the T3 ganglion location.^3^ We used this fact to compile the locations of T2 and T3 ganglia on the sympathetic trunk by dissecting human cadavers. Using the technique of locating the ganglion by visualizing the white RC and its sympathetic trunk intersection, a precise T3 ganglionectomy ETS procedure was performed on pediatric patients (age <20 years) using only cutting mode cautery.

## Patients and Methods

### Cadaveric Specimen Dissections

Forty embalmed cadaveric thoraces—25 adult female (age 53-100 years) and 15 adult male (age 50-95 years)—were procured. The costal and vertebral pleura were dissected to expose the sympathetic trunk and white and gray RC of the T1-T4 spinal levels. The location of each thoracic ganglion was determined by tracing the white RC to the corresponding ICN. For each T2 and T3 ganglia, the superior and inferior borders were measured in relation to the superior and inferior margins of the ribs 2-4. Each ganglion's superior and inferior borders were determined by noting the broadening of the trunk into a larger diameter ganglion. The measurements consisted of the distance in millimeters of the superior border of the T2 ganglion from the inferior border of rib 2, the inferior border of the T2 ganglia from the superior border of rib 3, the superior border of the T3 ganglion from the inferior border of rib 3, and the inferior border of the T3 ganglion from the superior border of rib 4. After identifying and measuring the ganglia, they were digitally photographed (Nikon D750).

### T3 Ganglionectomy ETS

The total number of pediatric patients who underwent bilateral T3 ganglion ETS during the study period (2000-2023) was 74. ETS was done using two 5-mm ports near the axilla: one for the 30**°** endoscope and the second for the hook cautery. General anesthesia and either single or double-lumen endotracheal intubations were used. Carbon dioxide insufflation at 5-10 mm Hg pressure deflated the lung further. Arms were abducted, and the torso was raised about 30**°** to allow the lung to descend downward. The pleura was opened between the third and fourth ribs lateral to the sympathetic trunk with a hook cautery set at 30 W pure cutting mode by hooking and elevating the pleura. The white RC arising from ICN 3 is identified and followed toward the sympathetic trunk. The white RC is anatomically located on the lateral side of the sympathetic trunk compared with the gray ramus on the ganglion's medial or posterior side. The T3 ganglion was disconnected from the sympathetic trunk using a hook cautery set at pure cutting mode at 30 W on the rostral and caudal ends of the T3 ganglion. Gray and white RC were similarly interrupted at their entry/exit points into the ganglion with cautery set at pure cutting mode. Coagulation mode cautery was never used, to avoid thermal injury to surrounding tissues and adjacent nerves.

All data were recorded using Microsoft Excel (Microsoft Corporation). Mean and standard deviation were analyzed using GraphPad Prism 7 (GraphPad Software).

## Results

**Figure 1:**
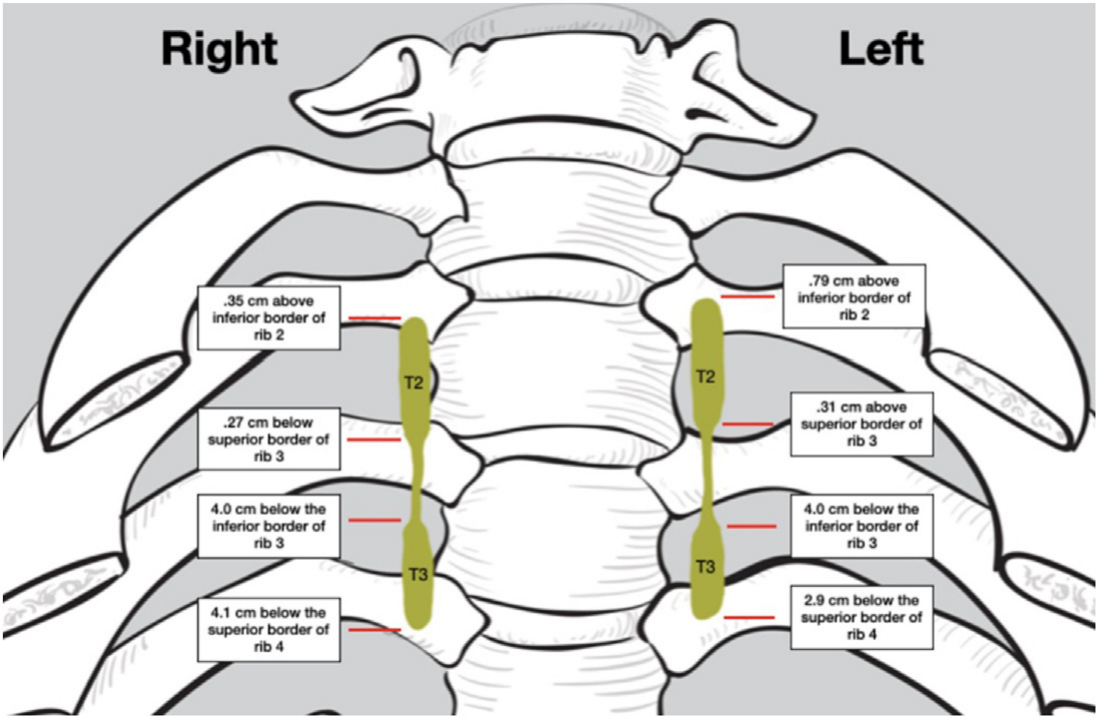
The data shown in the Table is illustrated in a picture form.

Bilateral dissection of 40 cadaveric specimens yielded 74 (37 right, 37 left) identifiable sympathetic trunks. Six (3 right, 3 left) dissections were discarded due to unacceptable dissection quality or unforeseen cadaver pathology. In the 74 accepted sympathetic trunks, all T2 and T3 ganglia were identified, resulting in 148 (74 T2, 74 T3) individual measurable ganglia. Each measurement was taken with a digital micrometer caliper. The data are shown in the Table. The following data are the critical findings: the mean inferior border of T2 ganglia was 0.27 mm inferior to the superior border of rib 3 on the right and 0.32 mm superior to the superior border of rib 3 on the left. This is the critical summary: 43% of the right T2 sympathetic ganglia and 38% of the left sympathetic ganglia had some contact with the superior part of the third rib. Figure 1 shows the Table data in picture form.

**Table:** Cadaver Dissection Results of T2 and T3 Ganglia Positions Relative to Ribs 2, 3, and 4

Result	Superior border of T2 ganglia from inferior border of rib 2 RIGHT	Superior border of T2 ganglia from inferior border of rib 2 LEFT	Inferior border of T2 ganglia from superior border of rib 3 RIGHT	Inferior border of T2 ganglia from superior border of rib 3 LEFT	Superior border of T3 ganglia from inferior border of rib 3 RIGHT	Superior border of T3 ganglia from inferior border of rib 3 LEFT	Inferior border of T3 ganglia from superior border of rib 4 RIGHT	Inferior border of T3 ganglia from superior border of rib 4 LEFT
Mean	+0.35	0.79	–0.27	+0.31	–4.0	–4.2	–4.1	–2.9
Max	+6.0	+11	+7.0	+9.0	+4.0	+3.0	+6.0	+8.0
Min	–3.0	–6.0	–8.0	–5.0	–14	–9.0	–18	–10
Median	+1.0	+1.0	0	0	–4.0	–4.5	–4.0	–3.0
SD	2.5	3.9	3.6	3.2	4.0	3.6	5.2	4.2

Values are presented in millimeters.

+/– in data refers to superior (+) or inferior (–) to the border of the indicated rib.

**Figure 2:**
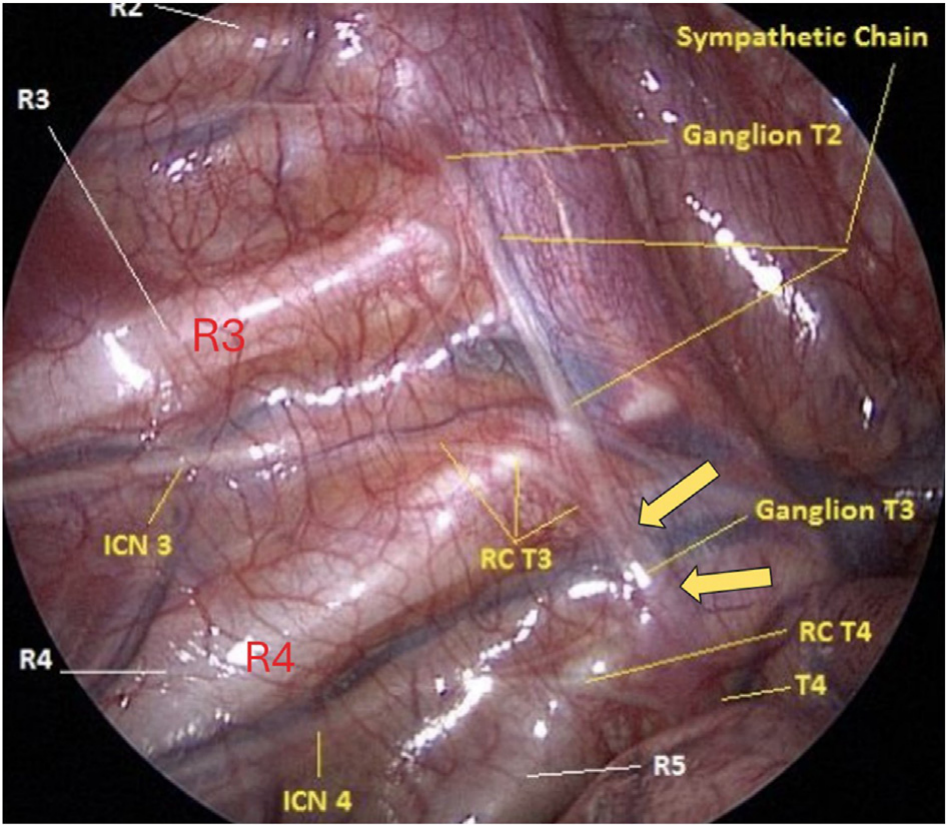
Intraoperative photograph shows the following structures: rib 2 (R2), rib 3 (R3), rib 4 (R4), rib 5 (R5), third intercostal nerve (INC 3), fourth intercostal nerve (INC 4), white ramus communicans connecting to T3 (RC T3), white ramus communicans connecting to T4 (RC T4), second thoracic sympathetic ganglion (Ganglion T2), third thoracic sympathetic ganglion (Ganglion T3), fourth thoracic sympathetic ganglion (T4). Note that white ramus communicans (RC T3) travel over the R4 and connect to the T3 ganglion in the R4 intercostal space. The large yellow arrows indicate the levels of rostral and caudal T3 ganglionectomy.

The mean age of the surgical patients was 17.1 years (range, 10-19 years). There were 44 male and 29 female patients. The total percentage of T3 ganglion overlying R4 was 65%. The total T3 ganglion position percentage in the third intercostal space was 35%. Figure 2 illustrates the positions of R3, R4, R5, ICN 3, white RC connecting to T3 (RC T3), and sympathetic trunk in a right thoracic trunk. The yellow arrows point to the upper and lower borders of T3, where the sympathetic trunk is cut.

Hyperhidrosis Disease Severity Scale score for all patients before their operation was 4 (“My sweating is intolerable and always interferes with my daily activities.”). The follow-up period for the patients was one year. All (100%) patients expressed satisfaction with their surgical outcome. No patient complained of compensatory sweating. There were no intraoperative complications.

## Comment

ETS is the treatment of choice for medically uncontrollable palmar hyperhidrosis. Due to the incongruency of surgical techniques and variable clinical results, The Society of Thoracic Surgeons published their expert consensus statements for treating hyperhidrosis in 2011.^1^ In the consensus paper, a rib-oriented approach was recommended based on the high variability of the ganglion anatomy and the problematic identification of the ganglia due to the presence of fat. The paper concluded that the optimal operation for palmar hyperhidrosis is the interruption of the sympathetic trunk at the top surface of rib 3 using electrocautery or the application of metal clips. The intrinsic problem with this rib-oriented approach is that the sympathetic trunk is interrupted at an approximate level; it is unclear if accidental thermal injury to a nearby ganglion may have occurred. It is, therefore, not surprising that despite the consensus statement's publication, the compensatory sweating complication rate did not significantly decrease.^4^

The RC that arise from intercostal nerves are not short, stubby connecting nerves, but they may travel long distances caudally or rostrally before connecting to the sympathetic trunk.^5^ The number of RC attached to a ganglion may number from 1 or 2 for T3 and T4 ganglia, but for T1 and T2 ganglia, the number of rami could be multiple. Without finding the junction of the RC to the sympathetic trunk, the locations of thoracic ganglia within the sympathetic trunk cannot be easily discerned. Our cadaver dissection data show that a significant percentage of T2 ganglion lies close to the superior border of R3 or on the R3 itself. We found that 43% of the right T2 ganglia and 38% of the left T2 ganglia are in contact with the superior part of R3. The T2 ganglia, therefore, can be injured if R3 level sympathectomy is performed, particularly if coagulation mode electrocautery is used, which is commonly done by many surgeons. Chung and associates^6^ also showed that T2 ganglions touch or lie directly over R3 35% of the time. If the sympathetic trunk is interrupted at the middle part of the third rib, there is a high probability that the T2 ganglion will be partially damaged. Consequently, if the target ganglion is T3, the sympathetic trunk should not be disconnected over rib 3 since, by doing so, the T2 ganglion may become injured, which will increase the chance of compensatory sweating.

The method to cut the sympathetic trunk should be carefully considered to optimize the outcome. A modern-day electrosurgery machine, which is used to interrupt the sympathetic trunk, offers 3 modes of cutting through tissue: pure cut, blend, or coagulation mode. In pure cut mode, a cautery unit performs much like a scalpel unless the power setting is too low, which can lead to soft tissue coagulation, so we set the power setting to 30 W. Although there is little control over bleeding when the pure cut mode is utilized, there is minimal thermal injury to the surrounding tissue.

In conclusion, due to the proximity of the T2 ganglion to the third rib, accidental thermal injury to the T2 ganglion may occur when third rib-oriented ETS is performed. A ganglion-oriented T3 ETS can be accomplished by using the white RC as a guide to accurately locate the T3 ganglion, ensuring precise T3 ganglionectomy and preserving T2 ganglion.
